# Agrobacterium species bacteraemia, Switzerland, 2008 to 2019: a molecular epidemiological study

**DOI:** 10.1186/s13756-022-01086-y

**Published:** 2022-03-09

**Authors:** Lisa Balmer, Helena M. B. Seth-Smith, Adrian Egli, Carlo Casanova, Andreas Kronenberg, Jacques Schrenzel, Jonas Marschall, Rami Sommerstein

**Affiliations:** 1 Department of Infectious Diseases, Bern University Hospital, Bern, Switzerland; 2 Clinical Bacteriology and Mycology, University Hospital Basel, Basel, Switzerland; 3 Applied Microbiology Research, Department of Biomedicine, University of Basel, Basel, Switzerland; 4 Institute for Infectious Diseases, University of Bern, Bern, Switzerland; 5 Bacteriology Laboratory and Genomic Research Laboratory, Service of Infectious Diseases and Service of Laboratory Medicine, Geneva University Hospitals, Geneva, Switzerland; 6 Division of Infectious Diseases, Department of Internal Medicine, Washington University School of Medicine, St. Louis, MO USA

**Keywords:** Agrobacterium species, Outbreak, WGS, Nosocomial

## Abstract

**Background:**

*Agrobacterium* spp. are infrequent agents of bloodstream infections linked to healthcare-associated outbreaks. However, it is unclear if outbreaks also occur across larger geographic areas. Triggered by two local clusters from putative point sources, our aim was to detect potential additional clusters in Switzerland.

**Methods:**

We performed a nationwide descriptive study of cases in Switzerland based on a prospective surveillance system (Swiss Centre for Antibiotic Resistance, anresis.ch), from 2008 to 2019. We identified patients with *Agrobacterium* spp. isolated from blood cultures and used a survey to collect clinical-epidemiological information and susceptibility testing results. We performed whole genome sequencing (WGS) of available clinical isolates and determined their relatedness by single nucleotide polymorphism (SNP) variant calling analysis.

**Results:**

We identified a total of 36 cases of *Agrobacterium* spp. from blood samples over 10 years. Beyond previously known local clusters, no new ones were identified. WGS-based typing was performed on 22 available isolates and showed no clonal relationships between newly identified isolates or to those from the known clusters, with all isolates outside these clusters being at least 50 SNPs apart.

**Conclusion and relevance:**

*Agrobacterium* spp. bacteraemia is infrequently detected and, given that it may be healthcare-associated and stem from a point source, occurrence of multiple episodes should entail an outbreak investigation. With the help of the national antimicrobial resistance surveillance system we identified multiple clinical cases of this rare pathogen but found no evidence by WGS that suggested a nation-wide outbreak.

**Graphical abstract:**

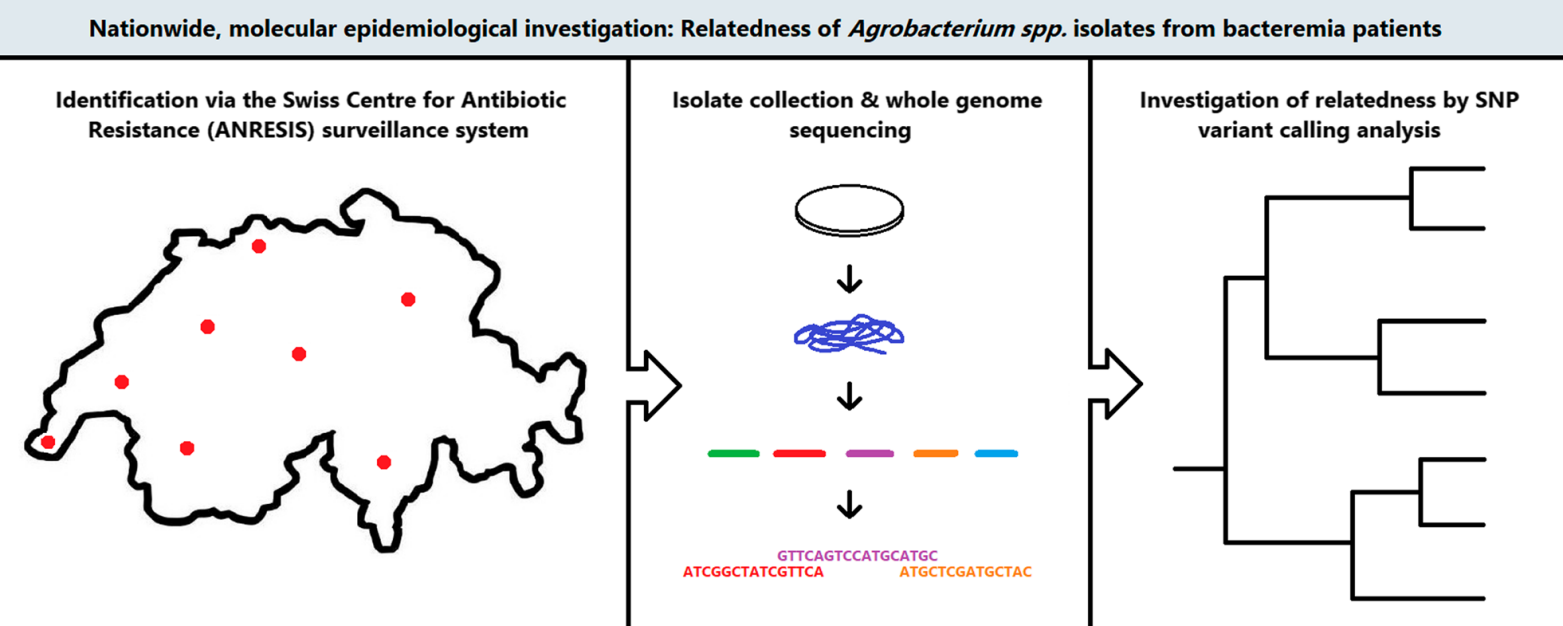

**Supplementary Information:**

The online version contains supplementary material available at 10.1186/s13756-022-01086-y.

## Introduction

The genus *Agrobacterium* is a group of gram-negative, aerobic and motile environmental bacteria. *Agrobacterium* species are recognized as rare opportunistic human pathogens, which affect mostly immunocompromised patients or patients with underlying diseases such as solid tumours or end-stage renal disease [1–4]. *A. pusense* has been described as the main human pathogen in the genus [5]. The majority of reported cases with *Agrobacterium* spp. are bloodstream infections related to the use of central venous catheters (CVC) [2–4, 6] or other permanent medical devices and foreign materials. In some cases, cure was only achieved by removal of the catheter [3, 4, 7]. This suggests that device colonisation plays an important role in the pathogenesis of bloodstream infection, which is supported by the ability of these bacteria to attach to silicone tubes [7] and the high colony counts found in catheter blood cultures [3]. Healthcare-associated cases have been reported [2, 6] and common infection sources for cases of *Agrobacterium* spp. bloodstream infections have been suggested previously [6]. On the other hand, community-acquired cases have been described as well [1, 3, 4] and in one study, a pulse-field gel electrophoresis of a cluster of cases showed distinct isolates, thus ruling out nosocomial spread [2]. Also, pseudo-bacteraemia with *A. radiobacter* (formerly named *Rhizobium radiobacter* [8]) due to contamination of blood cultures by an environmental source has been described [9]. A recent report from Brazil described strains of *A. radiobacter* as part of a three-species outbreak associated with the use of total parenteral nutrition and/or calcium gluconate [10].

From 2011–2017, a series of eight patients with *Agrobacterium spp.* bacteraemia was noted at Bern University Hospital [11]. All of these patients had previously undergone a CT scan and had received intravenous contrast medium. The relatedness of the corresponding isolates in two clusters (four *Agrobacterium* genomosp. 3 and two *A. radiobacter*) was confirmed by whole genome sequencing (WGS). This suggested a common transmission pathway with introduction from two different point sources. Despite an extensive outbreak investigation, the sources could not be identified [11].

Our aim was to expand the investigation on bacteraemia isolates to other Swiss healthcare institutions. The objective was to find *Agrobacterium* spp. bacteraemia cases in Switzerland and explore their relatedness by a molecular epidemiological approach.

## Methods

### Study design and setting

The study was designed as a nationwide, descriptive case series in Switzerland, based on a uniform case definition and a prospective surveillance system.

### Preliminary data

Potential cases of *Agrobacterium* species and/or *Rhizobium* species isolated from blood cultures in Switzerland between January 2008 and December 2017 were identified via a query by the Swiss Centre for Antibiotic Resistance (anresis.ch). Anresis.ch is a national surveillance system that collects routine antibiotic resistance data as well as data on antibiotic usage. They maintain an antibiotic resistance database and inform the public about resistance trends on a regular basis. Currently, 30 microbiology laboratories across Switzerland and > 200 healthcare institutions contribute, covering approximately 80% of the annual hospitalisation days in Switzerland [12]. All participating laboratories performed antimicrobial susceptibility testing (AST) according to the Clinical Laboratory Standards Institute (CLSI) guidelines from 2004 to 2010. From 2011 to 2013, most of them switched to using the European Committee on Antimicrobial Susceptibility Testing (EUCAST) definitions [13–15]. Species identification was performed according to individual laboratory standard procedures, and these may have changed during the period covered in this analysis. For example, the microbiological laboratory at Bern University Hospital switched from 16S‐rRNA gene sequencing to matrix-assisted laser desorption ionisation-time-of- flight (MALDI‐TOF) mass‐spectrometry based identification during the study period.

The initial query included the following information: sample date, sample type, identification at the genus and species level, the responsible diagnostic microbiology laboratory, and (if available) the corresponding healthcare institution [16].

### Survey and cases

Based on the query, a survey (Additional file 1: Fig. S1) was sent to the microbiology laboratories from which the *Agrobacterium* (*Rhizobium)* species had been reported. By means of the survey we collected antimicrobial susceptibility results and inquired about the availability of the isolates for further analysis. The case definition for inclusion was a first (non-duplicate within 30 days) patient isolate from a blood culture with *Agrobacterium* or *Rhizobium* spp. and a valid response to the survey. Cases identified through anresis.ch but without a valid laboratory response were excluded. Data from anresis.ch covered ten years, from January 2008 until December 2017. We also included cases not identified via the query but reported independently to us by the laboratories; these could also be from the years 2018 and 2019.

For the descriptive analysis, *Agrobacterium* und *Rhizobium* spp. were grouped together, due to changes in nomenclature and classification over the years [5, 17]. The “intermediate” susceptibility category was considered as resistant. For the evaluation, each healthcare institution was anonymized by an index number and each laboratory by an index letter.

### Whole genome sequencing and SNP phylogeny

DNA was extracted from isolates using a DNA extraction robot (Qiacube, Qiagen). Libraries were prepared using NexteraFlex, sequenced PE150 on a NextSeq 500 Illumina sequencing platform. The mean coverage for each sample was in excess of 47x. Assemblies were generated using Unicycler v0.4.8 [18] and used in Genome-to-Genome Distance Calculator (GGDC) [19] for digital DNA:DNA hybridization (dDDH) species determination against the panel of reference isolates described [11] using 70% cutoff (formula 2). A comparison of genomes was generated in the Type (Strain) Genome Server (TYGS) [20]. For single nucleotide polymorphism (SNP) calling and phylogeny within species we used CLC workbench v12.0.3 with parameters that differed from the default as: variant calling with 10 × minimum coverage, 10 minimum count and 70% minimum frequency, and SNP tree creation with 10 × minimum coverage, 10% minimum coverage, 0 prune distance and including multi-nucleotide variants (MNVs). As a reference assembly for *A. pusense*, GCA_900013495 was downloaded from NCBI and fragmented into reads using SAMtools [21] wgsim. Mapping was also performed against plasmids pTi-SAKURA (accession number NC_002147.1), pRi1724 (NC_002575.1) and *A. pusense* assembly GCA_900013495. All data generated here was submitted to the European Nucleotide Archive under project number PRJEB37957 (https://www.ebi.ac.uk/ena/data/view/PRJEB37957).

### Study size and potential bias

The study size was determined and restricted by the number of reported cases. To address potential reporting bias in case of an incomplete anresis.ch query, the contacted laboratories were encouraged to report additional cases not identified by the query. To prevent a selection bias due to differences in nomenclature, we included all *Agrobacterium (Rhizobium)* spp. that were reported to anresis.ch in the selected period.

### Analysis and statistics

We used R for descriptive analysis and the graphs (R Foundation for Statistical Computing, Vienna, Austria) [22].

## Results

### Cases

**Fig. 1:**
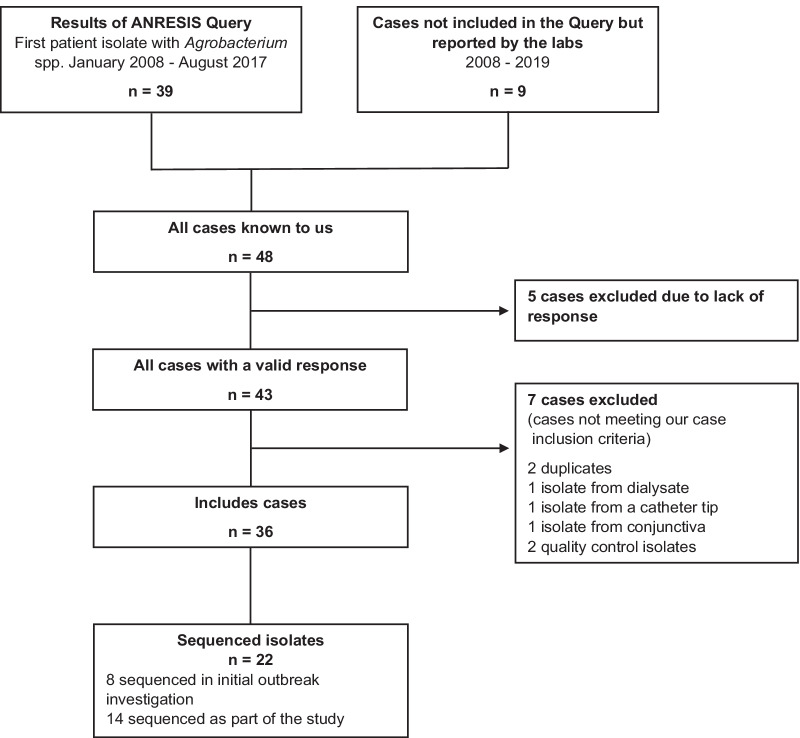
Flowchart of included cases and sequenced isolates

The initial nationwide query identified 39 cases of *Agrobacterium* or *Rhizobium* spp. from blood culture samples. Nine out of eleven contacted laboratories responded individually and reported nine additional cases not captured by the initial query. This resulted in 48 cases, from the years 2008 to 2019. We excluded twelve cases for not meeting the case definition; five with no response to the survey, three cases with *Agrobacterium* spp. from other sites (1 dialysate enriched in a blood culture bottle, 1 catheter tip, 1 conjunctival swab), two duplicate cases, and two quality control isolates. Our final dataset included 36 cases from ten healthcare institutions (Fig. 1).

**Fig. 2:**
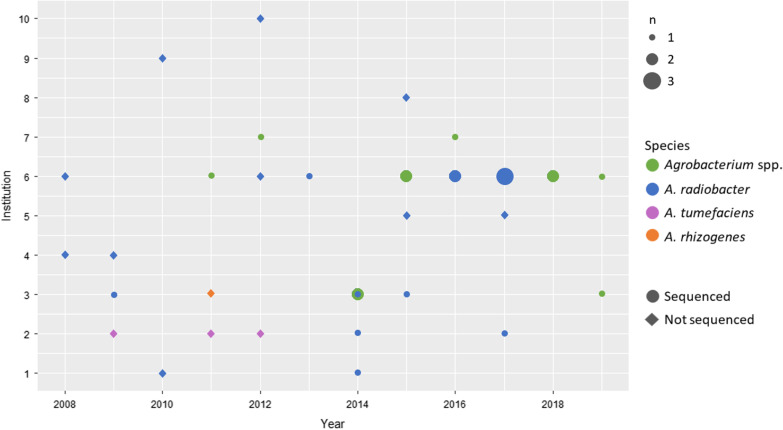
Overview of the 36 cases. We plotted the number of annual cases (x-axis) for all healthcare institutions (y-axis; 1–10) of *Agrobacterium* spp. bacteraemia and indicated availability for whole genome sequencing. Indicated are the initial presumptive species identifications by the originating microbiology laboratories, which may differ from the definite identification by WGS as shown in Fig. 3

An overview of the 36 cases according to annual cases per institution, initial species identification, and availability for WGS is shown in Fig. 2. According to the initial species identification by the originating microbiology laboratories, 22 (61%) were *A. radiobacter*, three were *A. tumefaciens,* and one was *A. rhizogenes*. In ten cases, the species was not identified beyond *Agrobacterium* spp..

We saw a temporal-spatial accumulation of cases only in Institution 6 (Bern University Hospital, where the first outbreak was described; Fig. 2). Of the other institutions with two or more reported cases, only one (Institution 3) reported more than one case in the same year. Of note, the two reported cases from Institution 1 stemmed from the same patient, who had two *A. radiobacter* bacteraemia episodes within four years. Unfortunately, only the latter of these two isolates was available for sequencing.

### WGS und SNP tree

**Fig. 3:**
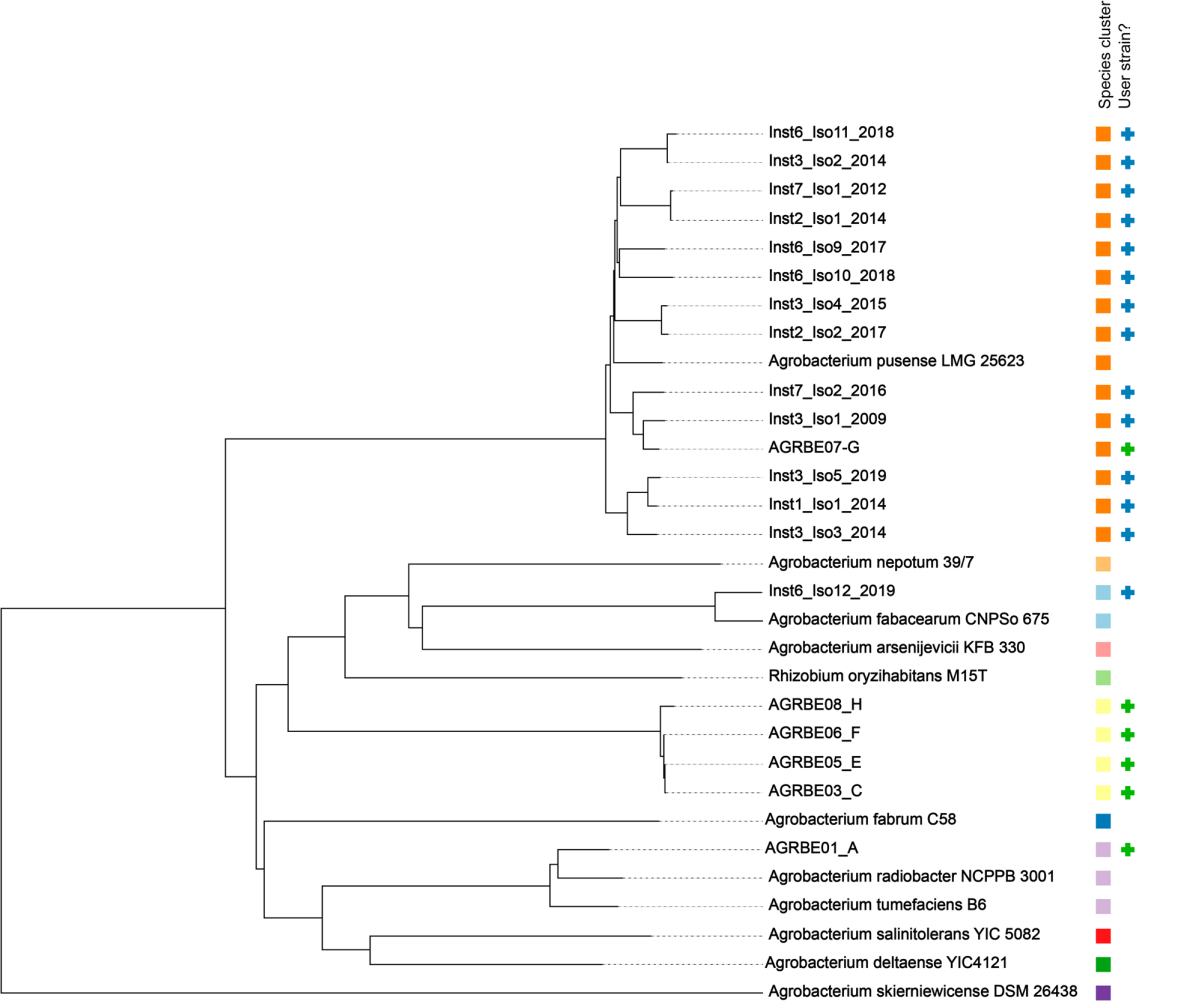
TYGS genome comparison of 20 of the 22 sequenced isolates compared to reference genomes. Isolates described in this paper are shown with blue + , including the previously described isolates [11] from the two clusters at Institution 6 (AGRBE03_C, AGRBE05_E & AGRBE06_F and ARGREBE01_A) with a green + . AGREBE02_B and AGRBE04_D were excluded from this analysis, as they are identical to AGREBE02_A and AGRBE04_C respectively. Species clusters are marked by colours, using 70% dDDH cutoffs

22 isolates from five healthcare institutions were sequenced (Fig. 2): eight from the initial outbreak investigations [11] and 14 additional isolates as part of this study. This accounts for 61% (22/36) of the cases included. Definite species identification by genomic comparisons (dDDH) against reference genomes showed that 13 of the isolates are *A. pusense*, and one *Agrobacterium* genomospecies 1, in addition to the previously described *Agrobacterium* genomospecies 3 (n = 5), *A. radiobacter* (n = 2) and *A. pusense* (n = 1) isolates [11]. A genome comparison shows the relationships between isolates and reference genomes (Fig. 3). Of the isolates belonging to *A. pusense*, several pairs of isolates, from different institutes in all cases, clustered together. A SNP comparison within *A. pusense* shows all strains differ from each other by over 900 SNPs with the exceptions of pairs Inst3_Iso4_2015 / Inst2_Iso2_2017 (53 SNPs in this analysis) and Inst7_Iso1_2012 and Inst2_Iso1_2014 (63 SNPs). None of the newly analysed cases are related to those from the outbreak that was described first (AGRBE03_C, AGRBE04_D, AGRBE05_E and AGRBE06_F) [11]. Mapping against pRi and pTi plasmids shows that these are not present in any of the isolates (< 27% and < 18% of the references covered by mapping reads, respectively).

### Susceptibility testing

Of the 36 cases included, AST data was available for 29. Most isolates were resistant to tobramycin (88%) and ceftazidime (43%), followed by trimethoprim-sulfamethoxazole (33%) and ampicillin (29%). The isolates were uniformly susceptible to carbapenems (imipenem and/or meropenem) and all but one were susceptible to fluoroquinolones (ciprofloxacin and/or levofloxacin). Further, some of the isolates with WGS-confirmed clonal relatedness showed variation in susceptibility. A summary of the susceptibility testing is shown in Table 1 susceptibility testing.

**Table 1:** Susceptibility testing

Antibiotic (isolates tested)	Sensitive (%)	Resistent (%)
Ampicillin, n = 17	71	29
Ceftazidime, n = 23	57	43
Cefuroxime, n = 16	81	19
Tobramycin, n = 17	12	88
Any Carbapenemes, n = 26	100	0
Any Fluoroquinolones, n = 29	97	3
Trimethoprim- Sulfamethoxazole, n = 15	67	33

Antimicrobial susceptibility of 29 of the 36 included *Agrobacterium* spp. isolates, as reported by the participating laboratories

Not all isolates were tested against all antibiotic agents

r, resistant; s, susceptible

## Discussion

### Key results

We identified 36 cases of *Agrobacterium* spp. from blood samples. Besides the previously published clusters [11], six further healthcare institutions reported more than one case over the investigated time period. A WGS-based typing of 22 isolates showed no close relationship between any of these cases, besides the previously established clusters [11]. Further, none of the newly sequenced cases were related to the 2013–17 clusters from Bern. In studies on clinical isolates of *Agrobacterium* spp. in other parts of the world, pulse-field gel electrophoresis and/or multilocus sequence-based phylogeny was used to analyse the relatedness of the strains [2, 5, 6, 23], therefore our WGS approach is unique and novel.

### Limitations

Our study size was restricted: not all healthcare institutions or microbiology laboratories report to anresis.ch, not all contacted laboratories responded to the survey, and not all the isolates were available for sequencing. Therefore, it is possible that not all cases that occurred in Switzerland during the study period were included.

Conventional methods (16S rDNA, API®, MALDI-TOF) used in clinical laboratories cannot reliably identify the species within the *Agrobacterium* genus and some commercial systems have predominantly or exclusively "*Agrobacterium/Rhizobium radiobacter*" in the database [11, 23]. Some isolates may thus have been initially missed and/or misidentified. Also, the database of the national surveillance does not include environmental samples and therefore such were not included in our study.

So far, no standardized AST breakpoints have been issued for *Agrobacterium* spp. EUCAST, however, publishes guidelines for groups of organisms, for which there are no established breakpoints. These guidelines state pharmacokinetic-pharmacodynamic (PK-PD) non species-related breakpoints should be used if available for the antimicrobial agent in question and that reporting “susceptible”, “intermediate” or “resistant” should be avoided for agents for which there are none; rather, the minimum inhibitory concentration (MIC) should be reported in those instances [24]. This lack of standardization complicates the comparison of susceptibility tests stemming from different laboratories. Further, the lack of standardization in susceptibility testing is a general limitation for outbreak investigations and an additional argument in favor of more sophisticated molecular analyses to assess relatedness of isolates.

### Clustering and WGS

Seven out of the ten healthcare institutions reported more than one case during the covered time period, which could suggest local clusters derived from a common transmission pathway or source. Such a persistent point source may be plausible even though cases from different centres were separated by months or years in time. The WGS and the following SNP variant calling analysis were able to rule out the transmission of any of the newly sequenced cases, as most showed no close relationship to each other, or to the previously known clusters. Of those differing by < 100 SNPs, they were from different institutions and separated by two years. However, we cannot exclude relatedness and potential past outbreaks in the cases that were unavailable for sequencing, for example the three cases of presumptive "*A. tumefaciens"* from Institution 2 between 2009 and 2012 and the ten presumptive "*A. radiobacter"* isolates across seven institutions. Nevertheless, we have strong evidence that a cross-institutional outbreak is unlikely.

### Antimicrobial susceptibility

The most frequent resistances we found were against tobramycin and ceftazidime. This matches case reports and case series of *A. radiobacter* bacteraemia published to date [3, 7, 25]. Like other studies, we also found variable susceptibilities to cephalosporins (ceftazidime and cefuroxime) [1, 2]. The almost uniform susceptibility of the isolates to carbapenems and fluoroquinolones is in line with published data [2–4, 7, 25].

Considering the lack in standardization in AST for this genus, the results of the susceptibility testing should be interpreted with caution, especially when comparing results from different laboratories or institutions as well as comparing them to findings from other studies. In addition, most laboratories switched from CLSI to EUCAST guidelines during the study period, so results from the same laboratory but from different years might also not be comparable.

The encountered variation in susceptibility between clonal isolates could also be explained with the technical difficulties in AST for these organisms. This underlines the limits of using susceptibility reporting to investigate relatedness of strains.

## Conclusion

We identified 36 cases of *Agrobacterium* spp. bacteraemia in Switzerland from 2008 to 2019. The strains were mostly resistant to tobramycin and ceftazidime and susceptible to carbapenems and fluoroquinolones. Besides the two established clusters at Institution 6/Bern University Hospital [11], six further healthcare institutions reported multiple cases. A WGS-based typing of the 22 isolates available showed no close relatedness between any of the cases, besides the previously established outbreak [11]. Thus, we conclude that nosocomial outbreaks of *Agrobacterium* spp. bacteraemias from a point source may occur but remain the exception. If multiple cases of invasive *Agrobacterium* spp. with the same species occur at one healthcare institution this should prompt an outbreak investigation. Suspicion should be raised in particular if case patients underwent the same procedure in the same location, as common transmissions pathways with introduction from persistent point sources, which may remain unrecognized for years, are possible.

With the help of the nation-wide surveillance system, we identified multiple cases of a rare pathogen and WGS revealed they were all unrelated.

## Supplementary Information

**Additional file 1**. Survey.

## Data Availability

All data generated was submitted to the European Nucleotide Archive under project number PRJEB37957 (https://www.ebi.ac.uk/ena/data/view/PRJEB37957).

## Abbreviations

Anresis.ch: Swiss Centre for Antibiotic Resistance
AST: Antimicrobial susceptibility testing
CLSI: Clinical Laboratory Standards Institute
CVC: Central venous catheters
dDDH: Digital DNA:DNA hybridization
EUCAST: European Committee on Antimicrobial Susceptibility Testing
GGDC: Genome-to-Genome Distance Calculator
MALDI‐TOF: Matrix-assisted laser desorption ionisation-time-of- flight
MIC: Minimum inhibitory concentration
MNVs: Multi-nucleotide variants
PK-PD: Pharmacokinetic-pharmacodynamic
SNP: Single nucleotide polymorphism
TYGS: Type (Strain) Genome Server
WGS: Whole genome sequencing

## Declarations

### Transparency declaration

None to declare.
