# Supplementary material for: Agrobacterium species bacteraemia, Switzerland, 2008 to 2019: a molecular epidemiological study
Source: Antimicrob Resist Infect Control. 2022 Mar 9;11:47. doi: 10.1186/s13756-022-01086-y (PMC8908629; doi:10.1186/s13756-022-01086-y)

# Supplementary material

# Supplementary Figure 1

**Survey**

As sent to the individual laboratories containing their cases.


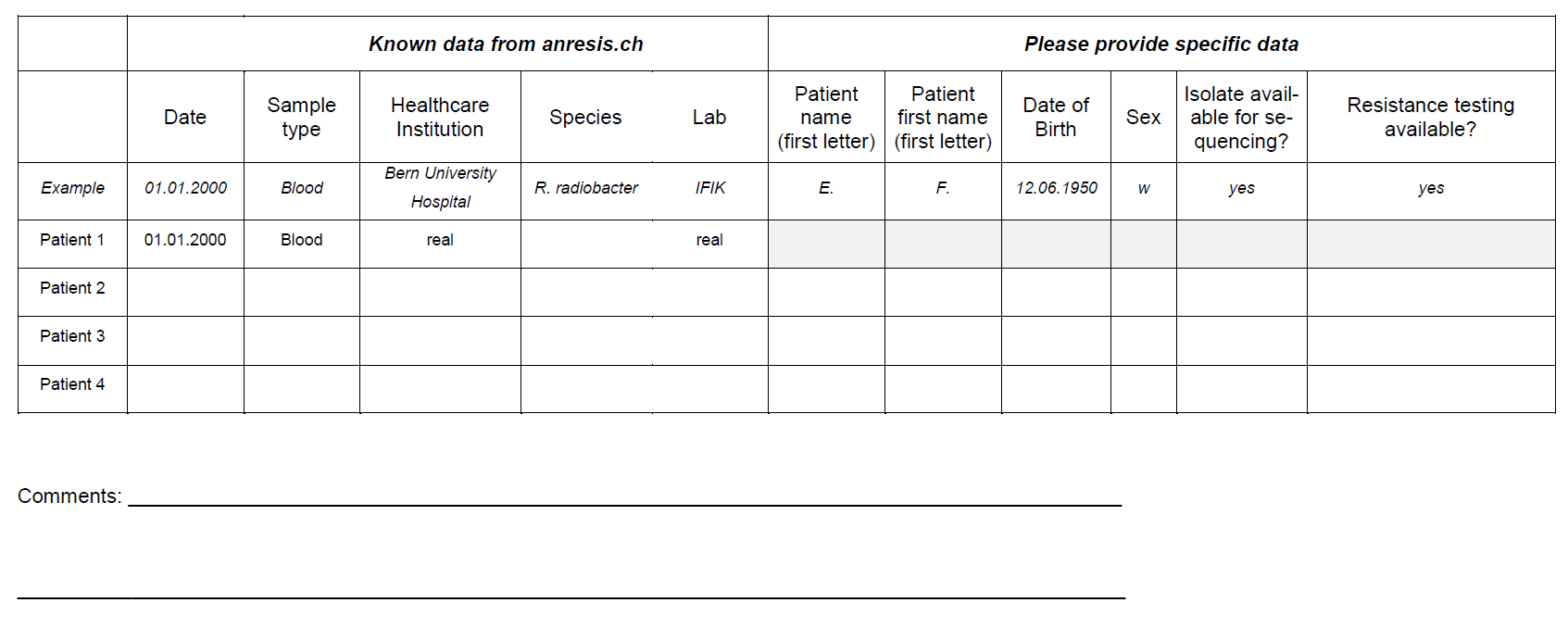

Supplement: Supplementary file 1 — Additional file 1. Survey. [file 13756_2022_1086_MOESM1_ESM.docx]
